# 

**Published:** 1880-12

**Authors:** 


DECEMBER, 1880.

### THE AMERICAN JOURNAL OF DENTAL SCIENCE,

NOTHING TO INDEX

EDITED BY

F. J. S. GORGAS, M. D., D. D. S.,

AND

JAMES B. HODGKIN, D. D. S.

VOL. 14.—THIRD SERIES.—No. 8.

BALTIMORE:

SNOWDEN & COWMAN.

LONDON:

TRUBNER & CO., 60 PATERNOSTER ROW.

1880.

PUBLISHED MONTHLY.

$2.50 PER ANNUM.

PAYABLE IN ADVANCE.

